# Vertical transport and plant uptake of nanoparticles in a soil mesocosm experiment

**DOI:** 10.1186/s12951-016-0191-z

**Published:** 2016-06-08

**Authors:** Alexander Gogos, Janine Moll, Florian Klingenfuss, Marcel van der Heijden, Fahmida Irin, Micah J. Green, Renato Zenobi, Thomas D. Bucheli

**Affiliations:** Agroscope, Institute for Sustainability Sciences ISS, 8046 Zurich, Switzerland; Department of Chemistry and Applied Biosciences, ETH Zurich, 8093 Zurich, Switzerland; Department of Chemical Engineering, Texas Tech University, Lubbock, TX USA; Artie McFerrin Department of Chemical Engineering, Texas A&M University, College Station, TX USA

**Keywords:** Nanomaterials, Black carbon, Soil leachate, Multi-angle light scattering, Microwave induced heating, Wheat, Red clover

## Abstract

**Background:**

Agricultural soils represent a potential sink for increasing amounts of different nanomaterials that nowadays inevitably enter the environment. Knowledge on the relation between their actual exposure concentrations and biological effects on crops and symbiotic organisms is therefore of high importance. In this part of a joint companion study, we describe the vertical translocation as well as plant uptake of three different titanium dioxide (nano-)particles (TiO_2_ NPs) and multi-walled carbon nanotubes (MWCNTs) within a pot experiment with homogenously spiked natural agricultural soil and two plant species (red clover and wheat).

**Results:**

TiO_2_ NPs exhibited limited mobility from soil to leachates and did not induce significant titanium uptake into both plant species, although average concentrations were doubled from 4 to 8 mg/kg Ti at the highest exposures. While the mobility of MWCNTs in soil was limited as well, microwave-induced heating suggested MWCNT-plant uptake independent of the exposure concentration.

**Conclusions:**

Quantification of actual exposure concentrations with a series of analytical methods confirmed nominal ones in soil mesocosms with red clover and wheat and pointed to low mobility and limited plant uptake of titanium dioxide nanoparticles and carbon nanotubes.

**Electronic supplementary material:**

The online version of this article (doi:10.1186/s12951-016-0191-z) contains supplementary material, which is available to authorized users.

## Background

It is scientifically ascertained that, due to their increased production and use, nanomaterials (NMs) will inevitably enter the environment [1], including soils. The currently most produced NMs are titanium dioxide nanoparticles (TiO_2_ NPs) [2]. They are used in diverse applications such as paints, UV-protection, photovoltaics and photocatalysis [3], but also as a food additive [4]. Carbon nanotubes (CNTs) are closing the gap in the last years, with 10-fold increased production volumes since 2006 [5]. Due to their extraordinary mechanical and electrical properties, CNTs are mostly used as building blocks in light-weight composite materials as well as electronics.

These particles can enter soils via different pathways [1, 6]. Application of biosolids to landfills and irrigation with surface waters is most likely for TiO_2_ NPs, while CNTs may enter soils via landfills and atmospheric deposition [7]. These types of release are unintentional, however, also applications in plant protection and fertilization have been foreseen [8, 9], which may lead to severely increased fluxes of these NP into soils. Apart from the positive effects and functions that are envisioned for agricultural applications of TiO_2_ NPs and CNTs [8, 9], such as protection of active ingredients and increased plant growth, respectively, also negative effects on microorganisms and plants have been reported [10–12].

The enduring uncertainty regarding the environmental safety of NMs highlights the need for a thorough risk assessment of these materials, which includes the study of their effects on organisms and the ecosystem as well as their fate. However, the analysis of NMs such as TiO_2_ and CNTs in complex systems such as real soils is challenging in many ways. For both, elemental analysis alone is not sufficient to trace the particles due to high elemental background concentrations of Ti and carbon.

Therefore, most studies until now used simplified laboratory systems as well as specifically labeled particles for eased detection to investigate both NM transport through porous media as well as plant uptake, often without confirmation of actual exposure concentrations. For example, TiO_2_ NP transport was investigated in sand columns under well controlled conditions [13, 14]. Fang et al. [15] studied TiO_2_ NP transport through soil columns at very high concentrations (40 g/kg). However, vertical translocation of both TiO_2_ NPs and CNTs has neither been investigated yet in large pot experiments or field studies, nor in the presence of plants. Plant uptake was shown for TiO_2_ NP in hydroponic exposure systems at high concentrations [16, 17]. In contrast, in a more realistic exposure setting using natural soil amended with TiO_2_ NPs, Du et al. [12] found no uptake of Ti into wheat. Also, CNTs were shown to be taken up into plants [18–20] from hydroponic systems. However, until now, no data is available for CNT uptake from natural soils, in which CNT transport and subsequent availability to plants could be different due to their high interactions with the soil matrix [21–23].

Here, we investigated the vertical distribution and leaching behavior of three different TiO_2_ (nano-)particles [P25, E171 and a non-nanomaterial TiO_2_ (NNM TiO_2_)] and the vertical distribution of a multi-walled CNT (MWCNT) within two elaborate pot exposure studies with red clover (*Trifolium pratense*) [24] and spring wheat (*Triticum spp.*) [25] in natural soil, and quantified their fractions in aboveground parts of the plants. We used recently developed methods such as microwave induced heating (MIH) [26] and asymmetric flow field-flow fractionation coupled to multi-angle light scattering (aF4-MALS) [27] to detect and quantify unlabeled MWCNTs in plant and soil samples, respectively. We additionally imaged root cross sections of exposed plants using (scanning) transmission electron microscopy. All data from this study were gathered to accompany two corresponding effect studies with actual, rather than nominal exposure concentrations. These studies examined the functionality of an agricultural ecosystem in presence of the NMs with regard to nitrogen fixation by the red clover-rhizobium symbiosis, as well as root colonization by arbuscular mycorrhizal fungi of both red clover [24] and wheat [25].

## Methods

### Chemicals and nanoparticles

Food grade E171 TiO_2_ particles were obtained from Sachtleben Chemie GmbH (Duisburg, Germany). All other chemicals and TiO_2_ nanoparticles were purchased from Sigma-Aldrich (Buchs, Switzerland). Uncoated titanium containing NPs were selected to represent different primary particle size ranges; average primary particle sizes were determined by TEM image analysis and were 29 ± 9 (P25, n = 92), 92 ± 31 (E171, n = 52) and 145 ± 46 nm (NNM TiO_2_, n = 49), see also Additional file 1: Figure S1. Anatase was the dominating crystal structure in all of the used particles. However, P25 also contains 20 % rutile, according to the manufacturer.

Multi-walled carbon nanotubes were purchased from Cheap Tubes Inc. (Brattleboro, VT). They were declared to have a length of 10–30 μm, and outer diameter of 20–30 nm, a purity of >95 % and an elemental carbon content of >98 %. The MWCNTs were used as received without further purification. Further characterization of the MWCNTs used was carried out and described in [27, 28]. All parameters were confirmed to be within the specified ranges with the exception of CNT length. The latter could only be determined in suspension, where it may have been altered due to sonication necessary for dispersing the particles.

### Soil

A natural soil was collected from an agricultural field at the facility of Agroscope, Zurich (N47° 25′ 39.564″ E8° 31′ 20.04″). The soil was classified as brown earth with a sandy loamy to loamy fine fraction. The top layer (5 cm) of the soil was removed and approximately 0.9 m^3^ of the underlying 15 cm topsoil were sampled. The soil was then sieved <5 mm, homogenized by shoveling it three times from one soil pile to another, and stored in a dry place until it was used in both red clover and wheat experiments.

### Spiking of the soil with NPs

Particle concentrations were selected to represent potential agricultural exposure scenarios as well as analytically accessible and potentially toxicologically effective concentrations. In a potential agricultural exposure scenario, fluxes from pesticide or fertilizer formulations may range from several micrograms to grams of NMs per kilogram of soil, depending on the formulation [8]. Thus, low doses (1, 10 mg/kg) were included as well as high doses.

For the spiking process, the soil was firstly blended with quartz sand (50 % v/v) to facilitate the recovery of below-ground plant organs after harvest. The properties of the soil-sand mixture are listed in Table 1. First, 300 g of the sand-soil mixture were each mixed with (i) 0.03 g (wheat experiment only), 0.3 g (red clover experiment only), 3 and 30 g of TiO_2_ NPs (both experiments), and (ii) 90 mg and 88 g MWCNT powder (clover experiment only), each in a 500 mL glass bottle which was rotated in a powder mixer (Turbula^®^ T 2 F, Willy A. Bachofen AG, Basel, Switzerland) for 30 min. For P25 and MWCNTs, the highest particle amounts resulted in a volume too big for the glass bottles. Therefore, these were split in two and four aliquots, respectively, and each aliquot mixed with 300 g sand-soil mixture.

**Table 1:** Properties of the soil-quartz mixture (50:50 v/v) administered to the pots

	Value	StDev
Org. C %	0.55	0.03
CEC mmol+/kg	6	
CaCO_3_ %	2.6	
pH	7.7	
Max. WHC g H_2_O/g dry soil	0.308	
Sand %	86.1	0
Silt %	6.3	0
Clay %	6.7	0.5

Into a cement mixer, 30 kg (including the pre-mixture) of a fresh sand-soil mixture (50 % v/v) were added, to yield final nominal NP concentrations of 1, 10, 100 or 1000 mg/kg, respectively, for TiO_2_ NPs, and 3 or 2933 mg/kg for MWCNTs. The mixing chamber was covered with a plastic sheet to avoid dust formation and run for 6 h. The soil was not dried before mixing to avoid changes to the microbial community structure, also investigated in Moll et al. [25]. Actual exposure concentrations were verified by X-ray fluorescence spectroscopy (XRF, for TiO_2_) and chemo-thermal oxidation at 375 °C [28] [CTO-375, for MWCNTs/Black Carbon (BC)] analysis as described below.

### General experimental design

A detailed description of the general setup, design and execution of the underlying exposure experiments is given in [24, 25]. In brief, for each plant type seven pot replicates were generated for each NP treatment, consisting of seven plants per pot for red clover and three for wheat. Non-plant controls were not performed because these two studies were primarily designed to observe possible biological effects of the NP treatments. Each pot was filled with a drainage layer of sand (0.5 L, 520 g) and 3.3 kg soil (corresponding to 2.9 L). Each pot was kept at 50–60 % (wheat) and 60–70 % (red clover) of the total water holding capacity (WHC, Table 1) during the entire experiment. Plants were grown over a period of 14 weeks (red clover) and 12 weeks (wheat) in a greenhouse with a 16 h light period (light intensity of 300 W m^−2^) and a 25/16 °C light/dark temperature regime. Wheat plants were fertilized weekly starting after week 3. Red clover plants were fertilized after 6 and 9 weeks, respectively. The composition of the nutrient solutions is given in the Additional file 1.

### Sampling of soil cores

Soil cores were sampled at the day of harvest from each pot using a conventional soil driller with a 2 cm diameter. Two cores were taken per pot and each divided into three depths (0–5, 5–10 and 10–15 cm). For each depth, both subsamples were joined into one and stored in plastic bags at 4 °C until further processing.

### Titanium analysis in soils with XRF

The soil samples from the cores were dried at 60 °C until a constant weight resulted, and ground to a fine powder using a Retsch ZM400 Ball Mill (Retsch GmbH, Haan, Germany) with a tungsten carbide bead at a frequency of 25/s for 5 min. Four grams of ground soil were homogenously mixed with 0.9 g of wax and pressed to a 32 mm tablet at 15 tons. Tablets were analyzed using an energy-dispersive XRF spectrometer (XEPOS, SPECTRO Analytical Instruments GmbH, Kleve, Germany). For correction of matrix effects, standard additions of the respective material to the soil were performed. For quality assurance we also analyzed a certified lake sediment reference sample (LKSD1, CANMET Mining and Mineral Sciences Laboratories, Ontario, Canada) with recoveries for Ti of >95 %.

### Titanium analysis in leachates with ICP-OES

A week before harvest, each pot was watered with 520 mL tap water, leading to approx. 110 % WHC. Consequently, 45 mL of leachate were collected through a valve at the bottom of the pots. The leachate was analyzed on the same day without any further treatment using inductively-coupled plasma optical emission spectrometry (ICP-OES) (ARCOS, SPECTRO Analytical Instruments GmbH). For quality control, an external Ti containing standard solution (ICAL, Bernd Kraft GmbH, Duisburg, Germany) was analyzed. The instrumental limit of quantification for Ti was determined at 22 μg/L.

### MWCNT analysis of soil with CTO-375

The CTO-375 procedure used in this study is described in detail in Sobek and Bucheli [28] as well as specifically for this work in the Additional file 1. This method quantifies total soil BC, which also encompasses MWCNT-carbon. We analyzed the soil samples taken from the cores, as well as the bulk spiked soil before the experiment. For the latter, six random grab samples of approx. 10 g were taken from the spiked pile.

### MWCNT analysis of soil with aF4-MALS

The method for MWCNT detection using aF4-MALS is described in detail by Gogos et al. [27]. Briefly, 120 mg of dry and ground soil from the cores were extracted with 10 mL of a 2 % sodium deoxycholate/0.05 % sodium azide solution, sonicated three times for 10 min using a high power sonication bath (720 W, Bandelin, Switzerland) and centrifuged at 17,500 g for 10 min. The supernatant was then used as a working suspension. This procedure was performed for each replicate of each soil depth. Afterwards, the replicates of each depth were joined to form a collective sample and analyzed using aF4-MALS, which generates a shape factor ρ from the radius of gyration and the hydrodynamic radius for each time point in the aF4 fractogram. The difference in ρ (Δρ) compared to native soil is then used to detect the MWCNTs [27]. The method detection limit (MDL) of the present study is presented and further discussed in the “Results and discussion” section.

### Titanium analysis of plants with ICP-OES

Due to their high importance for agricultural scenarios, from both plants, the parts used as food or feed were analyzed, i.e. the whole aboveground red clover, and the wheat grains. Dried plant samples were ground to a fine powder using a Retsch ZM200 centrifugal mill (Retsch GmbH). Subsamples (100 mg) were digested in a mixture of 0.2 mL hydrofluoric acid, 1.5 mL nitric acid and 0.2 mL hydrogen peroxide using a microwave (Ultraclave, MLS, Germany). The sample volume was subsequently adjusted to 50 mL. Digested samples were analyzed using ICP-OES (CIROS, SPECTRO Analytical Instruments GmbH). For quality assurance we also analyzed an industrial sludge reference sample (standard reference material SRM 2782, NIST, Gaithersburg, US) with recoveries for Ti of >85 %.

### MWCNT analysis of plants with MIH

Dry plant material was ground to a fine powder as described before. The amount of MWCNT uptake was then quantified by MIH, which is described in detail by Irin et al. [26]. MWCNTs have a high microwave absorption capacity, which results in a rapid rise in temperature within a very short microwave exposure time. Original method development included the generation of a calibration curve using the thermal response as a function of known CNTs spiked into Alfalfa (*Medicago sativa)* root samples.

Utilizing the data from Irin et al. [26], a new calibration curve was generated, where the slope of the curve depends on the respective nanomaterial and the intercept on the sample type. To this end, first, the initial slope was corrected using a factor based on the ratio of the source nanomaterials (MWCNTs of this study) microwave sensitivity and the one of the Irin et al. study. The sensitivity was determined by exposing ~1 mg of MWCNT powder to 30 W microwave power (2.45 GHz frequency) and recording the final temperature rise immediately (within 1 s) with a temperature rise (∆T) of 346 °C. Second, the intercept was corrected based on the control plant microwave response. Additional file 1: Figure S2 shows the renormalized calibration curve for MWCNTs at 50 W (6 s). The plant samples from the controls and the two MWCNT treatments were then tested at 50 W over 6 s and the quantity of MWCNT uptake were calculated using this new calibration curve. The limit of detection (LOD) as well as the limit of quantification (LOQ) where calculated based on the temperature rise from five measurements of control plant samples (blank signal) according to Keith et al. [29] (3 and 10 σ above the blank signal, respectively).

### Transmission electron microscopy of root cross sections

Fresh root samples were washed with tap water and pre-fixed in 2.5 % glutaraldehyde in phosphate buffered saline directly on the day of harvest and stored at 4 °C until processing. Ultrathin cross Sects. (70 nm thickness) were obtained by cutting root samples embedded in epoxy-resin using an ultramicrotome (Ultracut E, Leica, Wetzlar, Germany). The detailed sample preparation steps are provided in the Additional file 1. Ultrathin sections were imaged using a TEM (Tecnai G2 Spirit, FEI, Hillsboro, USA), coupled to an energy-dispersive X-ray (EDX) spectroscope (X-Max, 80 mm^2^, Oxford Instruments, Abingdon, UK) as well as a STEM (HD-2700-Cs, Hitachi, Japan) coupled to an EDX system as well (EDAX, NJ).

### Statistics

In the case of normal distributed residuals and homogenous data, an analysis of variance (ANOVA) was applied. If these model assumptions were not fulfilled, a Mann–Whitney test was conducted. All statistical analyses were done with the software R (version 3.01, the R Foundation for Statistical Computing) integrated in RStudio (version 0.97.551, RStudio, Boston, MA).

## Results and discussion

### Vertical soil distribution and leaching of Ti

**Fig. 1:**
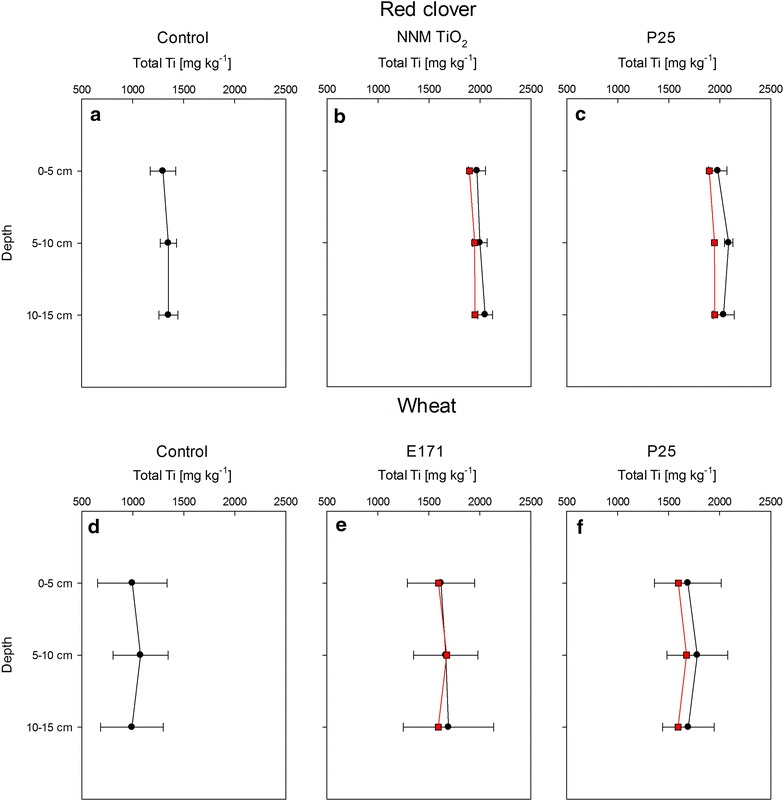
Vertical distributions of elemental Ti as determined by XRF analysis for three depths and for two different exposure experiments: **a**–**c** Red clover controls and red clover exposed to 1000 mg/kg of NNM TiO_2_ and P25 and **d**–**f** Wheat controls and wheat exposed to 1000 mg/kg of E171 and P25.* Error bars* show the standard deviation of seven replicates. *Red squares* show the predicted concentrations based on the control values and the nominal amount of Ti that was added as TiO_2_ NPs

Only the highest exposure concentration (1000 mg/kg) was analytically accessible using XRF, i.e., standard deviations among the replicates were in the order of the added Ti amount in samples spiked with <1000 mg/kg TiO_2_. Actual dry weight exposure concentrations of Ti were almost always slightly higher at the time of harvest than the initial nominal ones predicted from native and added Ti amounts, probably due to the residual water content in soils at the time of spiking (Fig. 1b, c, e, f). However, the differences were minimal (2.5–7.6 %) and overall not statistically significant (except for Fig. 1c, P25 1000 mg/kg, 5–10 cm), indicating that the employed spiking procedure was rather reliable. The control soils in the wheat experiment were systematically—though not significantly—lower in Ti content and showed higher standard deviations compared to the controls in the red clover experiment. This unexpected result may be explained by the fact that the two experiments were conducted independently using different subsets of the native soil and also highlights the necessity to verify actual exposure concentrations.

No statistically significant difference could be found between the different soil layers in any of the treatments (Fig. 1). Still, some trends could be observed; the distribution profiles of Ti in the control and in the P25 (80 % anatase, 20 % rutile) treatments were similar, with a tendency to slightly higher concentrations in the middle layer in both red clover and wheat pots. In contrast, the distribution profiles of the two pure anatase particles (NNM and E171) both tended towards elevated concentrations in the lowest part.

**Fig. 2:**
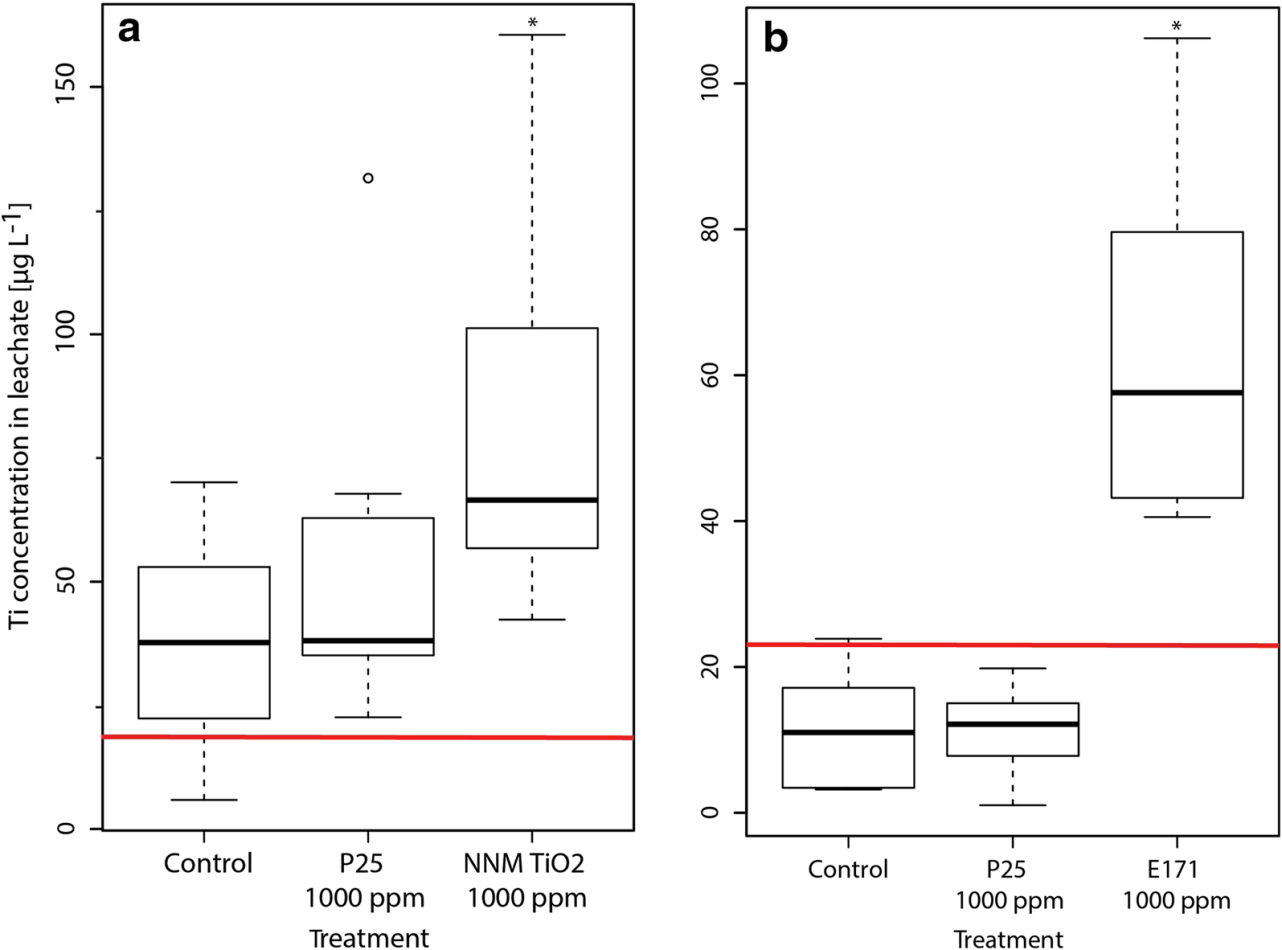
*Boxplots* (*solid line* = median) showing the Ti content of the leachates in the clover (**a**, each treatment n = 7) and wheat (**b**, each treatment n = 6) experiment. The LOQ is indicated with a *solid red line*. Significant difference (p < 0.05) of a treatment compared to the respective controls is indicated with an *asterisk*. The* lower* and* upper* borders of the *boxes* represent the 25th and 75th percentile, respectively. *Whiskers* represent maximum and minimum values, *circles* indicate outliers

In addition, Ti concentrations in leachates of these two treatments were significantly elevated compared to the controls (Fig. 2, p < 0.05), thus it can be assumed that the elevated Ti originated from eluting TiO_2_ NPs. However, the leached Ti amount—even in the treatments showing significantly higher concentrations—was very low and constituted not more than 10^−4^ % of the initial spiked Ti amount. In a dedicated transport study by Fang et al. [15], a soil with comparable properties (sandy loam, denoted as “JS soil”) showed a medium to high permeability for TiO_2_ NPs, attributed to the soil’s high sand content. A breakthrough of Ti in this soil started to occur after 1 pore volume. In our case, 520 mL of water was added to the pots (equivalent to 30 mm of precipitation) to collect the leachate, which correspond to 0.4 pore volumes only (1.24 L pore volume at full WHC). Thus, the added water amount was too low to initiate quantitative elution and would therefore explain the relatively low Ti concentration in the leachate after collection.

The observed difference in mobility (both in terms of Ti profiles and leachate content) may partly be explained by differences in the isoelectric point (IEP) of the TiO_2_ particles: while the more mobile NNM TiO_2_ and E171 exhibited a very low IEP of 2.2 (see Additional file 1: Figure S3), the one of P25 was 5.1, being much closer to the soil pH (7.7, see Table 1) and indicating a lesser colloidal stability [30]. TiO_2_ NPs with low IEPs may thus have a higher tendency to reach the groundwater and should thus be avoided in applications where this might be of relevance, e.g., when used as a component of a plant protection product [8, 9].

### Vertical soil distribution of BC/MWCNTs

**Fig. 3:**
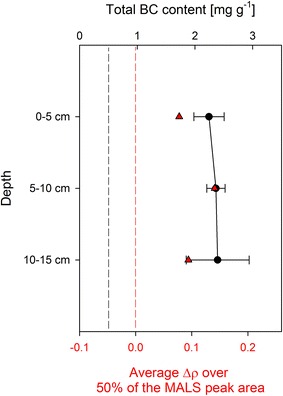
Vertical distribution of BC content in the *red clover pots*, determined by CTO-375 (*black circles*), and of Δρ values, indicative of the presence of MWCNT, determined by aF4-MALS (*red triangles*). The *dashed black line* shows the native BC content of the soil, while the *dashed red line* shows the MWCNT-free soil baseline in aF4-MALS (Δρ = 0). *Error bars* show the standard deviation of five replicates. Δρ values were determined once from pooled extracts of the five replicates

Figure 3 shows the BC distribution as well as the shape factor difference (Δρ) for the different soil depths of the 2933 mg/kg MWCNT amended red clover pots. As with Ti, only the highest MWCNT concentration was analytically accessible. The total background BC in the control soil was 0.50 ± 0.06 mg/g (n = 4). The specific recovery of the employed MWCNT in the soil over the CTO-375 method was 85 ± 13 % (n = 18, determined by standard addition). Therefore, the expected total BC concentration in the 2933 mg/kg MWCNT amended pots after CTO-375 can be calculated as follows: (2933 × 0.85) + 500 = 2993 mg/kg. However, the average BC content in the spiked soil before filling into the pots was lower than expected, with 2400 ± 100 mg/kg (n = 6), corresponding to 80 % of the expected BC concentration. Eventually, losses during the large scale mixing procedure could have contributed to these lower values. The variability of 4 % however suggests that the employed spiking procedure still resulted in a rather homogenous MWCNT distribution before the experiment. After the experiment, the average BC content quantified over all soil depths was 2330 ± 280 mg/kg, corresponding to 78 ± 12 % (n = 15) of the total expected BC concentration, with no significant difference between the layers. The average value was comparable to the BC content quantified before the experiment. However, precision, expressed by relative standard deviations, increased from 4 % (original spiked soil) to 12 % (aged soil). This increase in variability of the BC content may be associated with partial transport and/or aging (i.e. physiochemical modification of the particles, influencing their survival in CTO-375) of MWCNTs during the experiment.

To orthogonally observe the MWCNT behavior between the different layers with a second method, we also measured the cores with aF4-MALS [27]. With the soil of the present study, the MDL was at a Δρ of 0.099, corresponding to a CTO-determined MWCNT content of approx. 2 mg/g (Fig. 3), which is slightly lower than with the soil used in Gogos et al. (4 mg/g) [27]. The soil layers showed Δρ values of 0.078, 0.141 and 0.094 in descending order (Fig. 3). Thus, only the value of the middle layer was above the MDL. In combination with the results from CTO-375 and the increase in variability compared to the initial spike, this suggests a limited transport of the MWCNTs in the experiment. Such a low mobility would be in accordance to a dedicated soil transport study by Kasel et al. [22]. Using 14-C labeled functionalized MWCNTs, they found no detectable breakthrough in a comparable soil (loamy sand, denoted as “KAL” soil) even at water contents close to saturation (96 %).

### Plant uptake of Ti

**Fig. 4:**
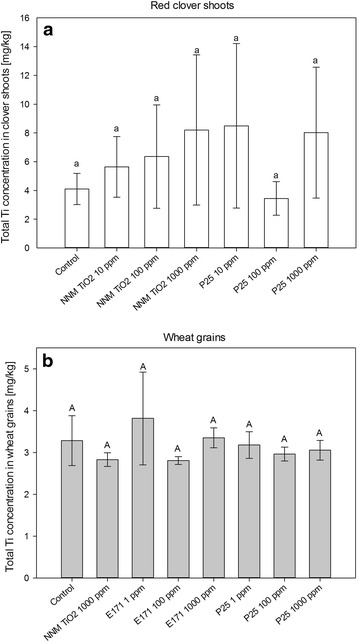
Total Ti concentration in red clover shoots (**a**) and wheat grains (**b**) for the different soil exposures. *Error* *bars* indicate one standard deviation (n = 4). *Different letters* above the *bars* indicate significant statistical difference (p < 0.05)

With 4.1 mg/kg, the determined Ti concentration in the red clover control plant material (Fig. 4a) was in the range of literature values for a plant species of the same family (*M. sativa*, a legume which also forms a symbiosis with rhizobia) and total soil Ti [31]. After treatment with TiO_2_ (nano-)particles, the average shoot Ti content of the red clover plants increased to 8 mg/kg at the highest exposure concentration of both NNM TiO_2_ and P25 (Fig. 4a). For NNM TiO_2_, the average Ti content was rising with the exposure concentration, whereas for P25 no such trend could be observed. However, variability within the treatments was relatively high, and no statistical difference between the different treatments was observed. Therefore, the Ti-content in the red clover plants was not dependent on a NNM or NM exposure.

**Fig. 5:**
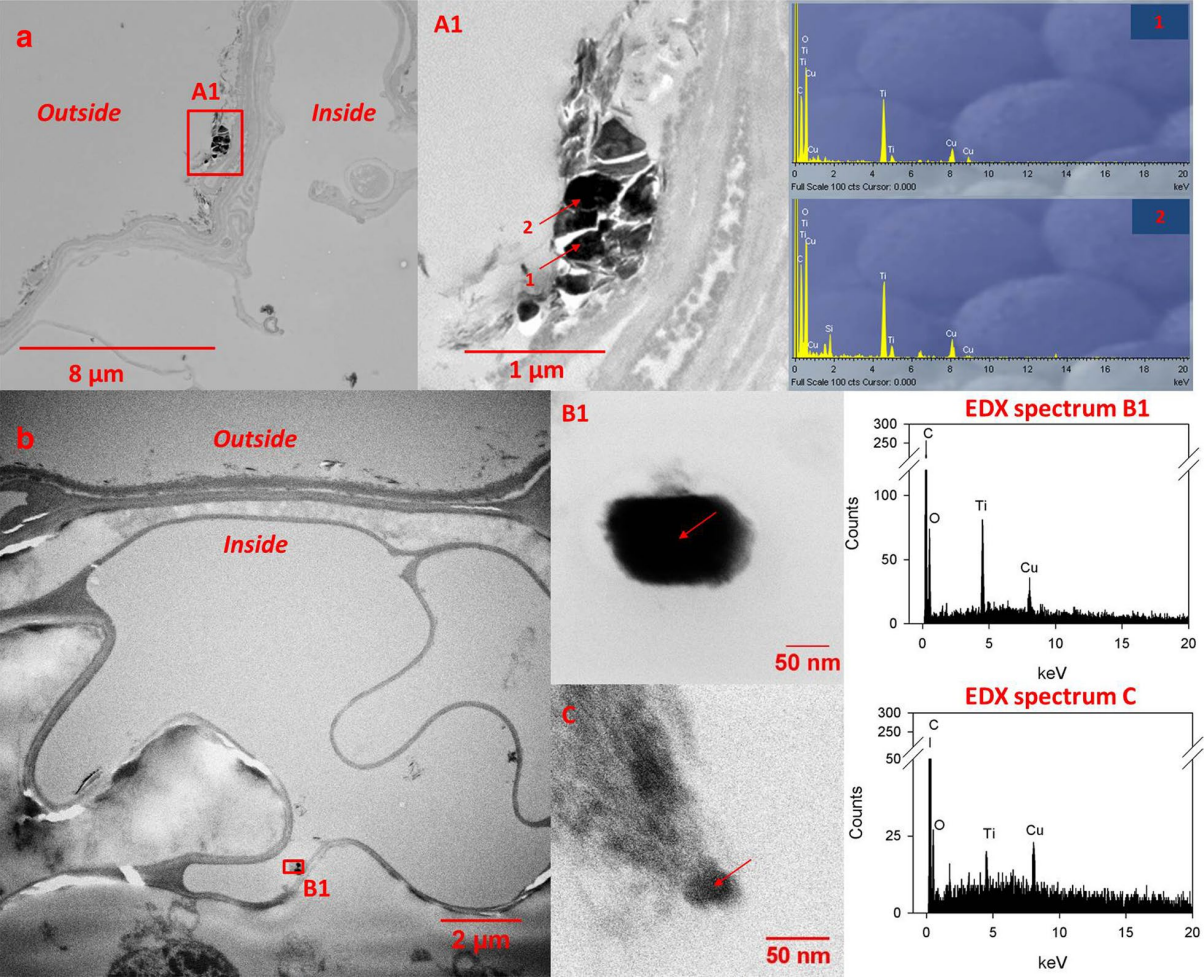
Electron microscopy micrographs of **a** an ultrathin-section of a root treated with 1000 mg/kg NNM TiO_2_ (imaged with TEM) together with a magnification (**A1**, outside of the root) and corresponding EDX spectra of selected spots (Spectrum 1 and 2) and **b** an ultrathin-section of a root treated with 1000 mg/kg P25 TiO_2_ (imaged with STEM) together with a magnification (**B1**, inside of the root) and corresponding EDX spectrum of the selected particle. **c** Represents a particle at a location different from **b**, but also inside a cell. EDX spectra were collected from the center of the particles. The copper (Cu) peak that is present in all spectra originates from the grid material

To elucidate whether the nevertheless elevated Ti contents within the red clover shoots was related to the uptake of actual TiO_2_ (nano-)particles, we investigated cross sections of these roots with TEM and EDX elemental analysis. In red clover roots treated with NNM TiO_2_, Ti containing particles with a similar morphology to the employed particles (Additional file 1: Figure S1A) were observed at the root surface (Fig. 5a, A1) but never inside the root cells. Some of these particles also contained Si (Fig. 5 A1, Particle 2) pointing to a possible natural origin of the particles. However, the absence of NNM TiO_2_ particles within the investigated thin sections does not necessarily disprove particle uptake, as it is not possible to representatively sample a whole plant root in this way.

In red clover roots treated with P25, only very few Ti containing nano-sized particles were found inside plant cells. The particle B1 in Fig. 5 shows a clear Ti EDX peak and is morphologically similar to the employed P25 particles (Additional file 1: elongated hexagon/Figure S1C). In addition, the oxygen peak in particle B1 is more distinct than in the other particles/objects, suggesting that the particle may consist of titanium-oxide/dioxide.

With an average of 3.3 mg/kg, the Ti content in the control wheat grains was slightly lower compared to red clover. In this case however, after treatment with TiO_2_ NPs, the average Ti content in the grains remained approx. constant (Fig. 4b). Thus, both for red clover shoots and wheat grains, no significant difference in Ti uptake between the different treatments and the controls could be found.

While no data is available for red clover plants, Larue et al. [32] and Servin et al. [16] demonstrated that nano-TiO_2_ can be taken up into wheat and cucumber, respectively, under extreme conditions (direct hydroponic exposure, high concentrations). Larue et al. [32] reported contents of up to 109 mg/kg Ti inside wheat roots, whereas Ti content in wheat leaves was below their LOD. To date, quantitative uptake data for aboveground plant material grown in natural TiO_2_ NP spiked soil however is available only from one study performed with wheat plants [12]. Therein, the Ti content of wheat grains was in the same range as in our study, with no significant uptake, confirming our observations. However, only one exposure concentration was employed (approx. 100 mg/kg TiO_2_ NPs), so no comparison can be made with regard to concentration dependent trends.

Altogether, our results suggest that Ti (-NP) uptake to red clover plants from real soils is insignificant. The biological data [24, 25] may represent another indirect piece of evidence, as for all endpoints (root and shoot biomass, number of flowers, nitrogen fixation and arbuscular mycorrhizal colonization), no significant effect of the treatments were observed for both plants.

### Plant uptake of MWCNTs

**Fig. 6:**
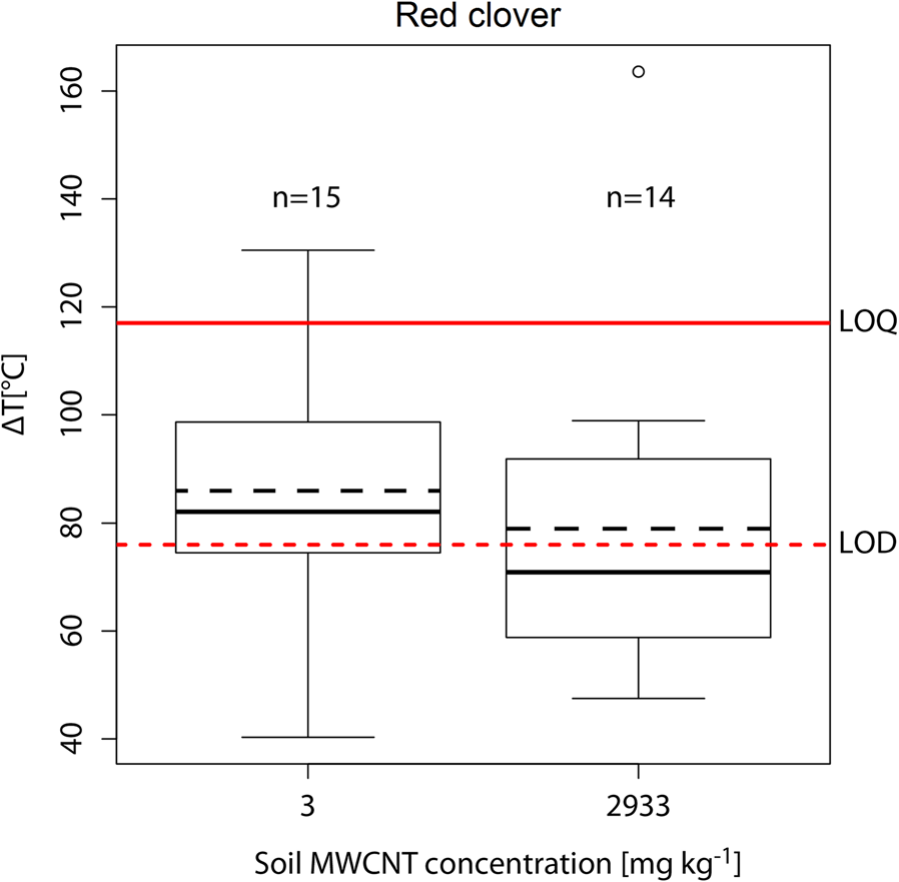
*Boxplots* (mean = *dashed*, median = *solid*) of the temperature increase at 50 W, 6 s for the red clover plant samples of the two MWCNT treatments (3 and 2933 mg/kg). The LOD (at ΔT = 76 °C, corresponding to 16 μg/g) is indicated with the *red dashed line* and the LOQ (at ΔT = 117 °C, corresponding to 55 μg/g) is indicated with the *solid red line*. Both LOD and LOQ have been determined 3 and 10 σ above the blank signal (control plants), respectively. All seven replicates of the treatments have been measured at least twice. The total number of measurements is indicated above the respective *boxplot*. The* lower* and* upper* borders of the *boxes* represent the 25th and 75th percentile, respectively. *Whiskers* represent maximum and minimum values

Figure 6 shows the temperature rise (ΔT, °C) of dry red clover shoot material from the two MWCNT treatments. The LOD of the MIH method [26] was calculated to be at ΔT = 76 °C (corresponding to a 16 μg/g MWCNT content) and the LOQ at ΔT = 117 °C (corresponding to a 55 μg/g MWCNT content).

A large fraction of the values was located in the region between LOD and LOQ, and can thus be considered as MWCNT detections (60 % of the values in case of the 3 mg/kg treatment and 43 % in case of the 2933 mg/kg treatment). The values above the LOQ represent MWCNT contents of 68 (3 mg/kg treatment, n = 1) and 99 μg/g (2933 mg/kg treatment, n = 1).

Taking into account the average dry weight of the red clover plants (14.3 g for the 3 mg/kg treatment and 15.3 g for the 2933 mg/kg treatment, see also Moll et al. [24]), the two cases with values above the LOD would correspond to a total amount of MWCNTs of 0.97 and 1.5 mg taken up into the plants per pot in the two treatments, respectively. This means that 9.8 % of the initial MWCNT amount in the soil would have been translocated to the shoots in the 3 mg/kg treatment. Conversely, in the 2933 mg/kg treatment, only 0.015 % of the initial amount would have been translocated. It is interesting to note that the MWCNT uptake was independent from the applied MWCNT concentration. In addition, we observed that within the MWCNT treatments, a significant reduction of flowering occurred (see Moll et al. [24]), which was not concentration dependent as well.

Uptake of CNTs into a plant cell is likely to be limited to the fraction dispersed in water. MWCNTs however are highly hydrophobic and prone to homo- as well as hetero-agglomeration with soil constituents. This in turn may result in a very small fraction of MWCNTs that remains well dispersed in the soil pore water. In addition, the plant surface may act as a filter that becomes clogged over time. However, further experiments are needed to explain this intriguing result.

We tried to orthogonally confirm the observed MWCNT uptake by using TEM imaging on cross sections of the plant roots. Khodakovskaya et al. [18] and Tripathi et al. [19] provided such optical evidence for CNT uptake from hydroponic solutions. However, in our case, the sole use of TEM was not conclusive. Additional file 1: Figure S4A shows a MWCNT-like particle that was observed within a plant root cell of the MWCNT treatment. This particle showed structural and dimensional similarity to the native MWCNTs administered to the pots (Additional file 1: Figure S4B). Still, this observation remained the only one within a number of cross sections that were manually inspected.

We then made additional attempts to screen the samples for the presence of MWCNTs with confocal Raman spectroscopy (Additional file 1: Figure S5). However, this approach requires that the sample is free (or almost free) of carbon allotropes (native carbon or contaminations), such as soot and amorphous carbon. In principle, Raman spectroscopy has enough sensitivity to detect single MWCNTs, but we observed that the spectra of MWCNTs and other carbon allotropes as well as cell wall material (i.e. lignin [33], which is present in clover roots [34]) had a large overlap which made the screening difficult.

While the exact amount of MWCNTs taken up could not be fully quantified and optical confirmation is still not entirely affirmed, based on the specificity of the MIH method, it is still suggested that MWCNTs were taken up and translocated to the aboveground part of the plant in some cases. Studies that reported plant uptake or cellular localization of CNTs until now were performed in hydroponic cultures, where the particles were freely available for interactions with the root [18, 19, 35, 36]. Uptake from soil would thus constitute a novelty; however, due to the lack of an orthogonal confirmation of the observed uptake, this result should be interpreted with care.

## Conclusions

In this part of a combined effect and exposure study we placed emphasis on a rigorous confirmation of actual NP exposure concentrations. To achieve this goal we applied an array of analytical techniques to the soil and plant samples, of which some are novel and used for the first time in this kind of effect studies. In particular, this includes the combination of CTO-375 and aF4-MALS that showed that MWCNTs exhibited a rather limited mobility in the soil, as well as MIH that showed a concentration independent uptake of MWCNTs into some plants. In addition, the battery of analytical techniques confirmed the relatively constant exposure situation in both TiO_2_ NP and MWCNT treatments over several months, with only subtle changes in concentrations, which could however be explained qualitatively with underlying NP/soil properties, distribution processes and experimental conditions.

## Additional file

10.1186/s12951-016-0191-z **Figure S1.** Bright field TEM micrographs and size information of TiO_2_ particles. **Figure S2.** MIH calibration curve. **Figure S3.** Dependence of the ζ-potential [mV] of TiO_2_ particles on pH. **Figure S4.** Transmission electron microscopy micrographs of a potential CNT structure. **Figure S5.** Raman spectra of the employed MWCNT powder and plant samples. **Text.** Composition of fertilizers, MWCNT analysis of soil with CTO-375. Detailed sample preparation steps of root cross sections for analysis using transmission electron microscopy.
