# Supplementary material for: Vertical transport and plant uptake of nanoparticles in a soil mesocosm experiment
Source: J Nanobiotechnology. 2016 Jun 8;14:40. doi: 10.1186/s12951-016-0191-z (PMC4897911; doi:10.1186/s12951-016-0191-z)
Supplement: Supplementary file 1 — 10.1186/s12951-016-0191-z Figure S1. Bright field TEM micrographs and size information of TiO2 particles. Figure S2. MIH calibration curve. Figure S3. Dependence of the ζ-potential [mV] of TiO2 particles on pH. Figure S4. Transmission electron microscopy micrographs of a potential CNT structure. Figure S5. Raman spectra of the employed MWCNT powder and plant samples. Text. Composition of fertilizers, MWCNT analysis of soil with CTO-375. Detailed sample preparation steps of root cross sections for analysis using transmission electron microscopy. [file 12951_2016_191_MOESM1_ESM.docx]

Supporting Information: Vertical transport and plant uptake of nanoparticles in a soil mesocosm experiment

Alexander Gogos^a,b^, Janine Moll^a^, Florian Klingenfuss^a^, Marcel van der Heijden^a^ , Fahmida Irin^c^, Micah J. Green^d^, Renato Zenobi^b^ and Thomas D. Bucheli^a,^*,

^a^Agroscope, Institute for Sustainability Sciences ISS, 8046 Zurich, Switzerland

^b^Department of Chemistry and Applied Biosciences, ETH Zurich, 8093 Zurich, Switzerland

^c^Department of Chemical Engineering, Texas Tech University, Lubbock, Texas, United States

^d^Artie McFerrin Department of Chemical Engineering,

Texas A&M University, College Station, Texas, United States

* Corresponding author. Tel.: +41 58 468 73 42; fax: +41 58 468 72 01; e-mail: thomas.bucheli@agroscope.admin.ch.


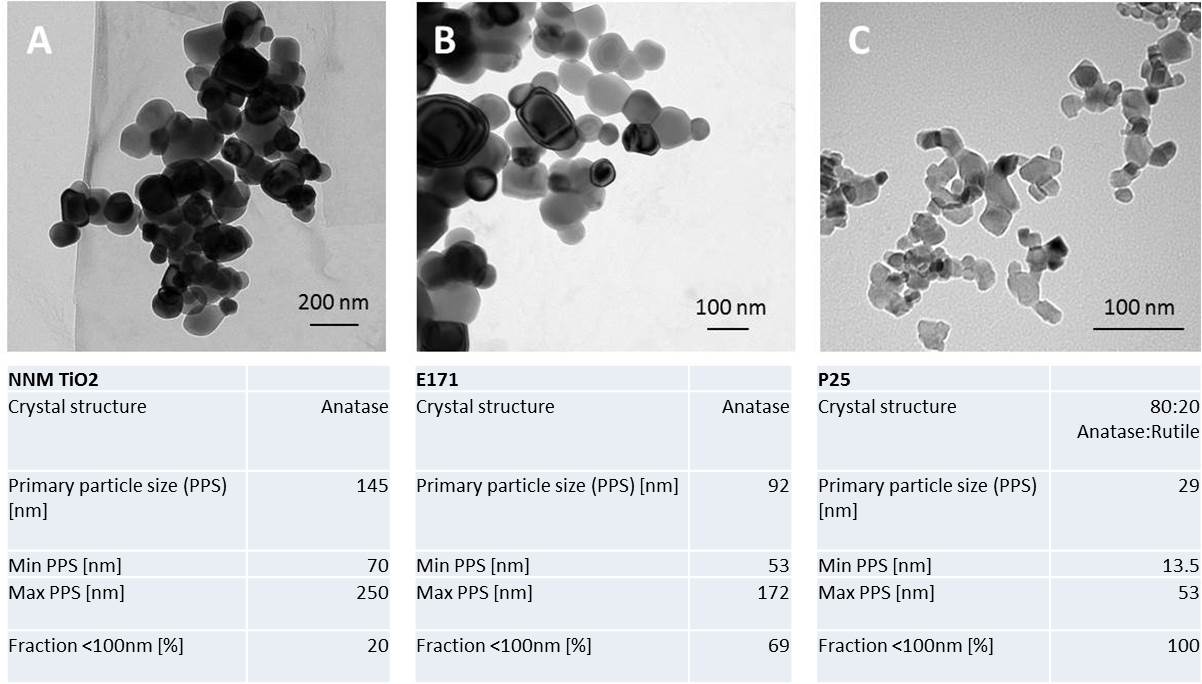


Figure S1 Bright field TEM micrographs of (A) NNM TiO_2_, (B) E171 TiO_2_ and (C) P25 particles, as used in the experiments together with corresponding size information determined by image analysis (longest distance through the particle).

**
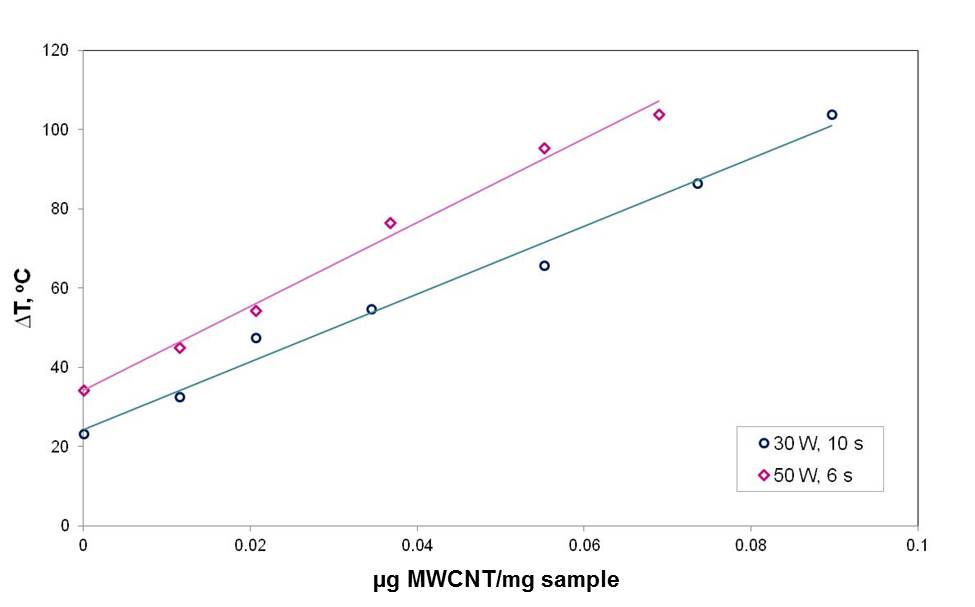
**

Figure S2 Calibration curve for the detection of MWCNT in plant samples. The curve is derived from the original calibration plot described in Irin et al.[[1](#_ENREF_1)] Note that the intercept of the curve varies with the heating behavior of the control sample whereas the slope remains constant for a given nanomaterial.


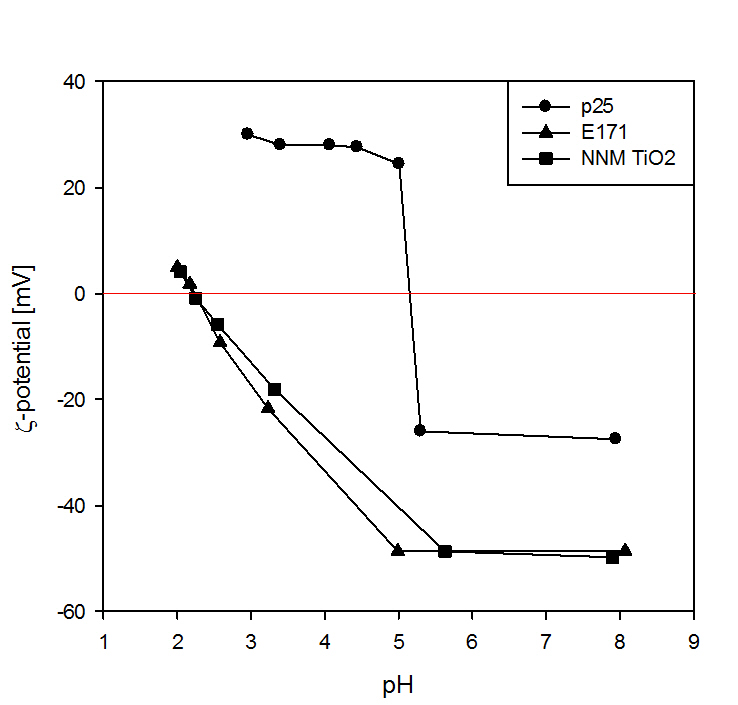


Figure S3 Dependence of the ζ-potential [mV] on pH for the used TiO_2_ particles. The dispersions were titrated in milli-Q water with hydrochloric acid/sodium hydroxide solution, respectively. Intercept with the red line (i.e. 0 mV) denotes the isoelectric point of the particles. Values represent the average of 3 measurements (n=3).


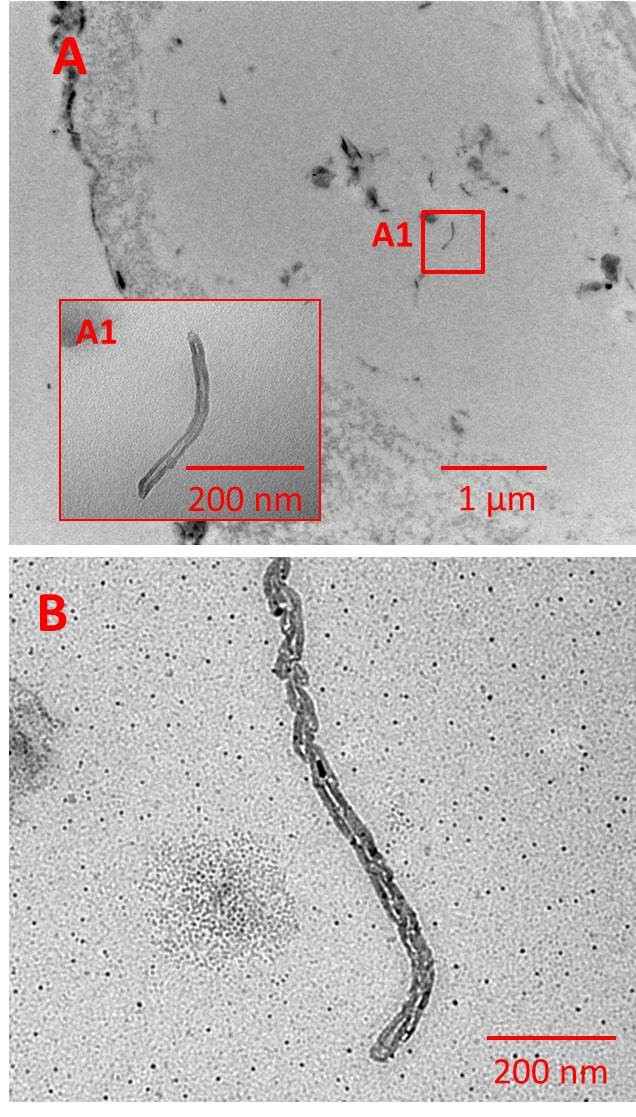


Figure S4 Transmission electron microscopy micrographs (A and B), showing (A) a potential CNT structure inside a red clover plant cell (epidermal cell, part magnified in A1) with structural and dimensional similarities to the MWCNTs administered to the pots (B).


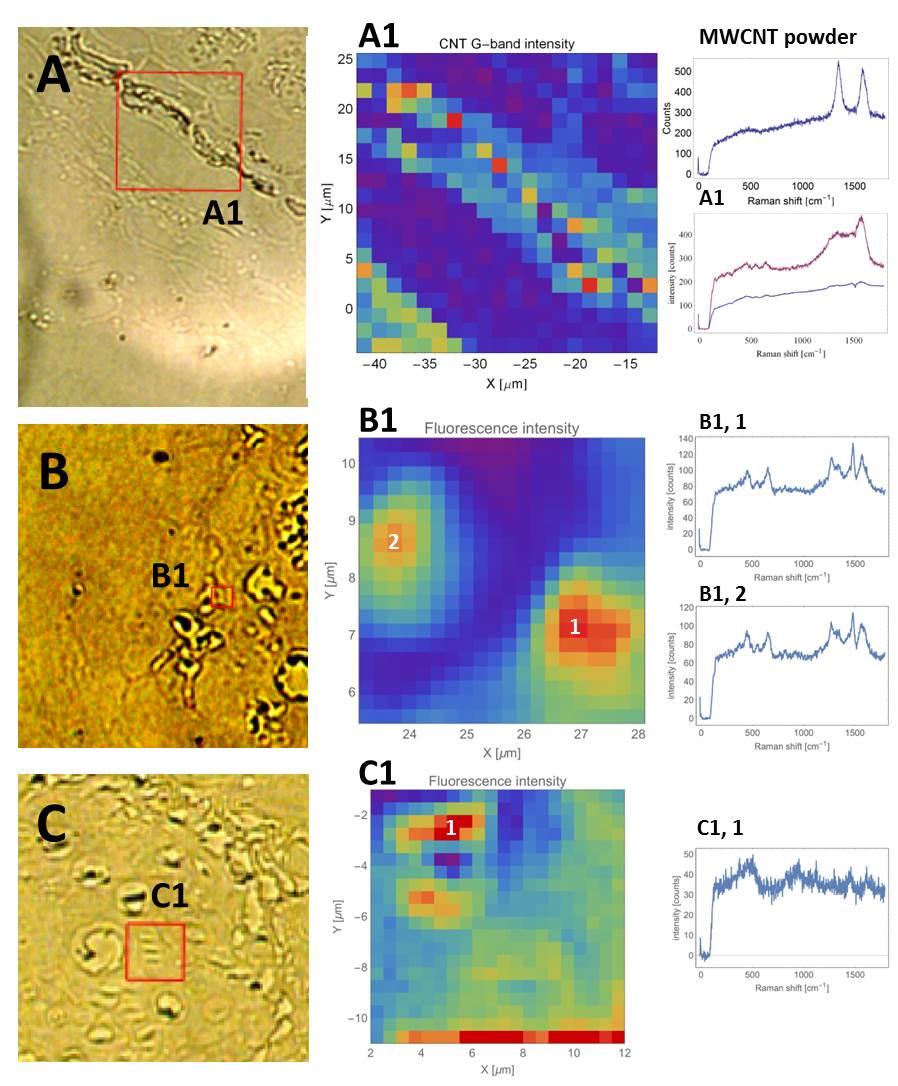


Figure S5 Optical images of root cross sections of controls (A and B) and a MWCNT treated red clover root (C). The regions indicated with a red square have been magnified for each optical image and been displayed as a Raman intensity map (A1, B1 and C1). The right panel shows the Raman spectrum of the employed MWCNT powder as well as Raman spectra taken from indicated regions of the corresponding Raman maps. The red spectrum in A1 corresponds to the highest intensity pixel, while the blue spectrum represents the average spectrum of the whole area. The spectra from B1 and C1 were taken from the indicated pixels.

**Composition of fertilizer solutions**

Wheat was fertilized every week, starting after week 3, with 7.9 ml of (KNO_3_ 60 mM, Ca(NO_3_)_2_·4H_2_O 40mM, NH_3_NH_4_ 7.5 mM, NH_4_H_2_PO_4_ 5 mM, MgSO_4_·7H_2_O 10 mM, in 990 ml water with addition of 10 ml micro nutrient solution (KCl 37 µM, H_3_BO_3_ 25 µM, MnSO_4_·H2O 2 µM, ZnSO_4_·7H_2_O 2 µM, CuSO_4_·5H_2_O 0.5 µM, (NH_4_)_6_Mo_7_O_27_·4H_2_O 0.5 µM, Fe(III) EDTA 20 µM)).

Clover was fertilized after 6 and 9 weeks with 10 ml of a solution containing KH_2_PO_4_ (5 mM), MgSO_4_·7H_2_O (1 mM), KCl (50 µM), H_3_BO_3_ (25 µM), MnSO_4_·H2O (1.3 µM), ZnSO_4_·7H_2_O (2 µM), CuSO_4_·5H_2_O (0.5 µM), (NH_4_)6Mo_7_O_27_·4H_2_O (0.5 µM), and Fe(III) EDTA (20 µM).

**MWCNT analysis of soil with CTO-375**

Briefly, all soil samples were dried at 105°C until weight constancy was achieved, and ground to a fine powder as described before. Dry and ground soil was then weighed into Ag-capsules, and subjected to thermal oxidation at 375°C under a constant air stream for 24 h. Subsequently, CTO treated samples were fumigated with concentrated HCl for 4 h, washed, dried, placed into Sn capsules and analyzed using an elemental analyser (Euro EA, Hekatech, Germany). For quality assurance, we also analyzed a marine sediment (standard reference material SRM 1941b, NIST, Gaithersburg, US) with a known BC content. The obtained values were within ±5% of the BC content of previous own measurements as well as compared to Hammes et al. [[2](#_ENREF_2)]. Further quality assurance measures are covered in the results and discussion section in the main manuscript.

**Detailed sample preparation steps** **of root cross sections for analysis using transmission electron microscopy**

Prior to the next steps of the fixation/embedding process, samples were rinsed three times with PBS. Samples were then incubated with 1% osmium tetroxide at room temperature for 40 min and then rinsed with water three times. Subsequently, samples were incubated with 1% uranyl acetate dihydrate in water for 1 h and rinsed with water three times. Dehydration was performed with 50% (15 min), 70% (20 min), 90% (25 min), 100% (5 min) and 100% water free ethanol for 30 min and propylene oxide 100% for 30 min.

Epon stock solution was prepared by mixing 70.89 g epon 812™, 92.35 g durcupan™ ACM and 8.68 g dibutylphthalate. For the working solution, 5.85 g epon stock solution were added to 5 g epoxy embedding medium hardener DDSA and 310 mg accelerator DMP 30. Samples were then incubated in 50% epon working solution and 2 times 100% epon working solution for 1h, respectively. The samples were polymerized at 60°C overnight.

The resulting epon blocks were pre-trimmed with a razor blade and mounted into and ultramicrotome (Ultracut E, Leica, Wetzlar, Germany). Ultrathin cross-sections of the roots (70 nm) were then cut using a diamond knife, transferred to a formvar/carbon coated copper TEM grid and dried at room temperature.

**References**

1. Irin F, Shrestha B, Canas JE, Saed MA, Green MJ. Detection of carbon nanotubes in biological samples through microwave-induced heating. Carbon. 2012;50(12):4441-9.

2. Hammes K, Schmidt MWI, Smernik RJ, Currie LA, Ball WP, Nguyen TH et al. Comparison of quantification methods to measure fire-derived (black/elemental) carbon in soils and sediments using reference materials from soil, water, sediment and the atmosphere. Global Biogeochem Cycl. 2007;21(3):GB3016.
